# QUAM-AFM: A Free Database for Molecular Identification by Atomic Force Microscopy

**DOI:** 10.1021/acs.jcim.1c01323

**Published:** 2022-03-02

**Authors:** Jaime Carracedo-Cosme, Carlos Romero-Muñiz, Pablo Pou, Rubén Pérez

**Affiliations:** † Quasar Science Resources S.L., Camino de las Ceudas 2, E-28232 Las Rozas de Madrid, Spain; ‡ Departamento de Física Teórica de la Materia Condensada, Universidad Autónoma de Madrid, E-28049 Madrid, Spain; § Departamento de Física Aplicada I, Universidad de Sevilla, E-41012 Seville, Spain; ∥ Condensed Matter Physics Center (IFIMAC), Universidad Autónoma de Madrid, E-28049 Madrid, Spain

## Abstract

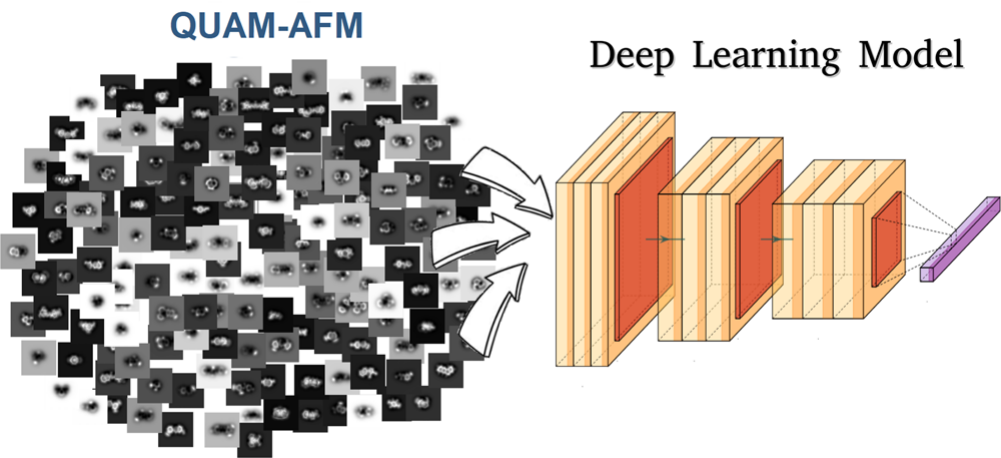

This paper introduces Quasar Science Resources–Autonomous University of Madrid atomic force microscopy image data set (QUAM-AFM), the largest data set of simulated atomic force microscopy (AFM) images generated from a selection of 685,513 molecules that span the most relevant bonding structures and chemical species in organic chemistry. QUAM-AFM contains, for each molecule, 24 3D image stacks, each consisting of constant-height images simulated for 10 tip–sample distances with a different combination of AFM operational parameters, resulting in a total of 165 million images with a resolution of 256 × 256 pixels. The 3D stacks are especially appropriate to tackle the goal of the chemical identification within AFM experiments by using deep learning techniques. The data provided for each molecule include, besides a set of AFM images, ball-and-stick depictions, IUPAC names, chemical formulas, atomic coordinates, and map of atom heights. In order to simplify the use of the collection as a source of information, we have developed a graphical user interface that allows the search for structures by CID number, IUPAC name, or chemical formula.

## Introduction

AFM has become one of the key tools for materials characterization and manipulation at the nanoscale. Operation in the frequency modulation AFM mode, commonly known as noncontact (NCAFM), in combination with the use of metal tips functionalized with a CO molecule (or other inert chemical species like a noble gas atom) at the tip apex has conferred this technique the ability of unveiling the internal structures of molecules with truly outstanding resolution.^1−4^ This experimental breakthrough has implications beyond the structure imaging as this unprecedented resolution has been extended to the study of the charge distribution in molecules,^5,6^ the discrimination of bond orders,^7^ the visualization of frontier orbitals,^2^ or even the tracking of chemical reactions on surfaces.^8−12^

This fast progress has been possible thanks to the combination of experimental and theoretical work. The CO-terminated tip AFM contrast is mainly controlled by the Pauli repulsion between the lone pair of the oxygen atom in the CO molecule and the charge density of the sample, modified by the interaction with the sample’s electrostatic field and enhanced by the probe tilting.^13−16^ The complex interplay of these interactions makes the interpretation of the experimental features highly nontrivial, and theoretical simulation methods are necessary in order to fully understand the experimentally recorded AFM images and to extract valuable information about the system under study. AFM simulation models with different complexities and accuracies^14,16−21^ have been developed to compute theoretical AFM images using as input the geometry of the molecule. These results have revealed the influence of the electrostatic force,^15,22^ the role of the CO–metal tip charge distribution,^21,23^ and the important contribution of both the short-range chemical interactions and electrostatics in the determination of bond orders and the imaging of intermolecular features on hydrogen and halogen bonded systems.^16,24,25^

A database of theoretical AFM images for a large collection of molecular structures, illustrating the AFM contrast associated with the most relevant chemical moieties in organic chemistry, can play a key role to support research efforts in different fields. AFM practitioners, for instance, could easily check the expected appearance of the AFM image of a particular molecule (or family or related molecules) under study and get insight into the correlation between the chemical properties of the system and the corresponding AFM fingerprints. The recent and fast growing efforts devoted to the development of on-surface synthesis^12^ can greatly benefit from such a database for the identification of the intermediates, final products, and reaction pathways.

Moreover, this data set can provide a robust and comprehensive reference for new artificial intelligence-based theoretical developments on scanning probe microscopy^26−29^ and, in particular, in AFM.^30−33^ Deep learning approaches have already been applied in AFM to the automated searching and identification of self-organized nanoparticle assemblies^30^ and in the case of high-resolution AFM images with CO-functionalized tips to obtain information on the molecular structure directly from a set of AFM images,^31^ to predict quantitative maps of the electrostatic potential,^33^ and to achieve automated chemical identification of 60 different molecular structures from both theoretical and experimental AFM images with a high accuracy.^32^ This last work clearly illustrates the huge amount of input data needed for the training of deep learning models. The solution of the relatively simple classification problem posed there required a data set of almost half a million AFM images.^32^ Considering the use of a combination of high-resolution AFM images and deep learning to achieve a complete chemical identification (structure and composition) of arbitrary molecules, the data should include a huge variety of chemical compounds, spanning all of the structures, chemical species, and functional groups relevant in organic chemistry. Building such a data set from experimental images would be completely unfeasible, given the amount of work involved in the experimental preparation of each individual molecular system. Conversely, simulations allow us to massively produce a huge number of theoretical AFM images fulfilling every necessary requirement to be used in the training of deep learning models.

Here, we describe the Quasar Science Resources–Autonomous University of Madrid atomic force microscopy image data set (QUAM-AFM), a user-friendly AFM image database, containing 685,513 molecules, that we have built in order to support research in on-surface synthesis and, particularly, deep learning strategies for molecular identification. Below, we focus on the discussion of the molecular structures included in the database and the choice of AFM operational parameters used to generate the theoretical AFM images that contains approximately 165 million gray-scale images. They have been calculated using an approximate version of the full density-based model (FDBM)^16^ implemented in the latest release^34^ of the probe particle model (PPM) suite of codes^14,18^ (see Methods). We have used the PubChem repository^35,36^ to extract the initial geometries of the molecules. We take advantage of this huge data bank for small molecules and use directly the CID number provided in PubChem to identify and label each molecule included in our database. AFM images are stored together with a set of descriptors that include the chemical composition, IUPAC name, or ball-and-stick representation of the molecule. The identification by CID number together with the distribution of the data in neat folders makes QUAM-AFM very accessible to train deep learning models. We have implemented a graphical user interface (GUI) which provides easy access to both the AFM images and the graphical descriptors of each molecule and allows a quick search by the CID number, composition, or IUPAC name. Although smaller AFM data sets have already been developed,^31−33^ our proposal aims to be the definitive reference in the field, thanks to the comprehensive collection of molecular structures, data organization, and consideration of all the necessary elements to train reliable and reproducible deep learning models.^37^

## Methods

### AFM Simulation Method

The AFM simulations of QUAM-AFM have been generated with an approximate implementation of FDBM,^16^ FDBM@PPM, that is available in the latest release of the PPM suite of codes.^34^ Basically, this implementation splits the total tip–sample interaction in four contributions: short range (SR), electrostatic (ES), van de Waals (vdW), and a harmonic contribution in the tilting angle that accounts for the CO flexibility
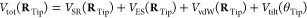
1where **R**_Tip_ is the position of the probe with respect to the sample, and θ_Tip_ the tilting angle of the CO with respect to the vertical direction of the simulation cell.

The van der Waals contribution, *V*_VdW_(**R**_Tip_), is calculated from the atomic geometry as an attractive contribution given by the *r*^–6^ term of the Lennard-Jones potential, as in the original PPM.^14,18^ In the FDBM, this contribution is calculated with an approach broadly used in density functional theory (DFT) calculations: the Grimme D3 semiempirical description of the dispersion interactions.^38^

The electrostatic (ES) interaction between the sample and the CO probe is calculated as the convolution of the sample total ES potential, *V*_sample_ (that includes the contribution of both electrons and ions) with a CO–tip multipolar electronic differential charge density Δ*ρ*
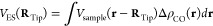
2Δ*ρ*_CO_ = ρ_CO_ – ρ_C_ – ρ_O_ is the difference between the electronic charge density of the molecule and the sum of the electronic charge densities of the C and O atoms. Both *V*_sample_ and Δ*ρ* are extracted from independent DFT calculations. Notice that, in the original FDBM, the total charge density of CO (including the contribution of electrons and ions) is used in the convolution, instead of Δ*ρ*_CO_.

The SR contribution, mainly Pauli repulsion between the close shell CO molecule and the sample, is defined through the overlap between the charge densities of the probe and the sample as
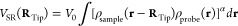
3where the parameters α and *V*_0_ have been set to 1 and 18 eV × Å^3^. Notice that this is not the case in the FDBM, where both α and *V*_0_ are used as fitting parameters to improve the matching with the DFT results.

**Figure 1:**
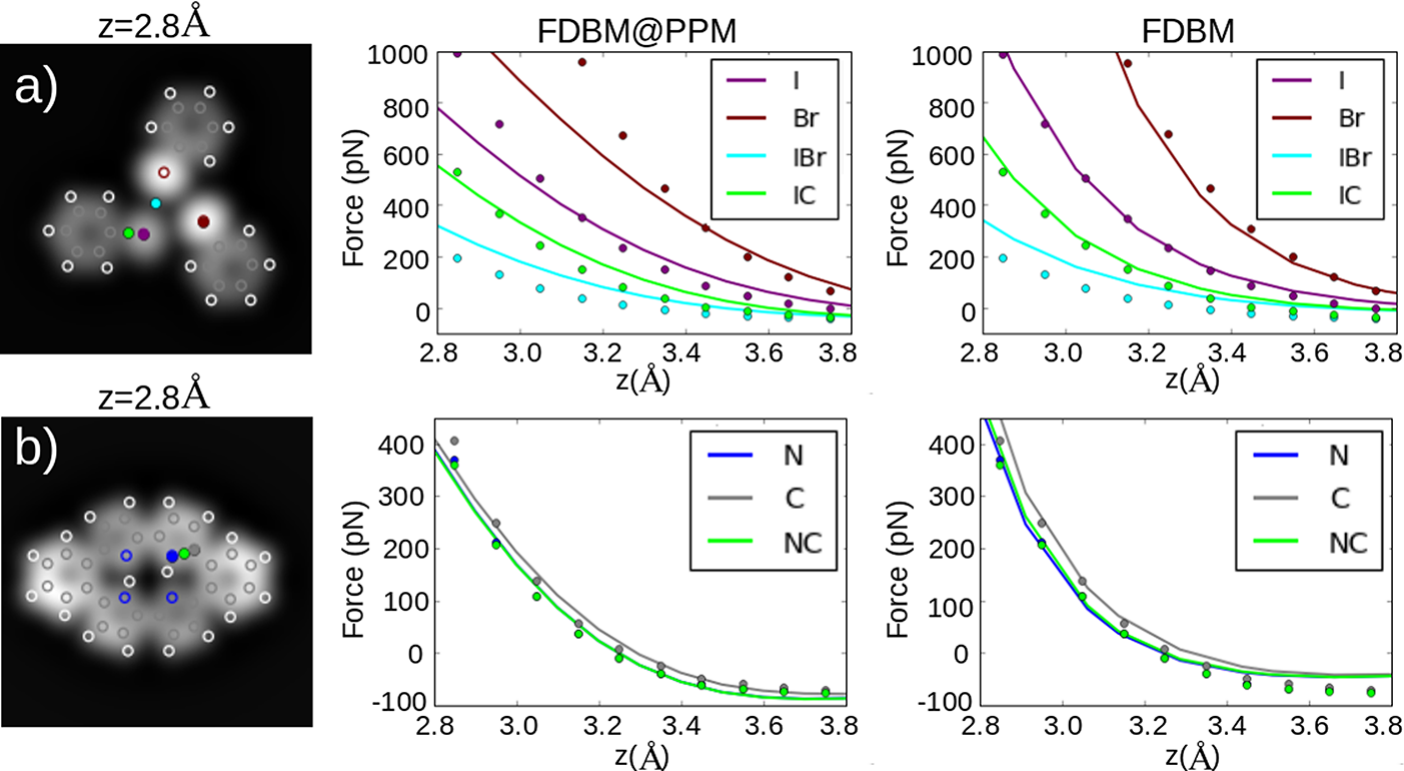
DFT force vs distance calculations probed by a CO molecule on (a) halogen bonded cluster^24^ and (b) meso-dibenzoporphycene (m-DBPc).^39^ No tilting of the CO molecule is allowed in these calculations. From left to right, scheme of the sites probed in the calculations, FDBM@PPM vs DFT forces, and FDBM vs DFT forces. DFT and model forces are represented by points and lines, respectively.

**Figure 2:**
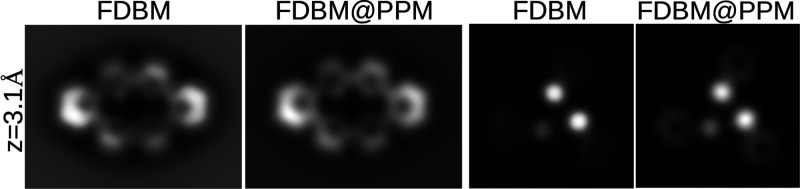
Comparison of static AFM force images (not including the CO tilting) at a relevant imaging distance (*z* = 3.1 Å) simulated with the FDBM and FDBM@PPM implementations described here for m-DBPc^39^ (left) and a halogen bonded cluster.^24^ This comparison shows that, in spite of the quantitative differences in the forces (see Figure 1), the FDBM@PPM implementation captures the relative contrast of the different sites and provides realistic AFM images.

Figure 1 compares DFT force calculations, not including the CO tilting, for two different molecules that include relevant chemical species like carbon, nitrogen, and halogen atoms, with the predictions of the FDBM (with the proper fitting of *V*_0_ and α) and the approximate FDBM@PPM implementation described above. The fitting of these parameters allows for a better match with DFT calculations in the relevant tip–sample distance range, where repulsive interactions dominate the contrast. The use of the overlap of the charge densities for the calculation of the SR interaction assures that the model reproduces the effects that different atomic species and chemical properties (like the bond order) introduce in the contrast observed in AFM images. The implementation used here provides accurate quantitative description of the forces and captures the relative contrast of the different sites, as further illustrated by the AFM images at the relevant imaging distance shown in Figure 2. These results confirm the effectiveness and reliability of this implementation to provide realistic AFM images while avoiding the unfeasible task of fitting the parameters for the huge number of molecules included in our database. This fact, together with the computational efficiency needed to build a huge database, and the possibility to efficiently generate frequency shift images for each molecular structure with different cantilever oscillation amplitudes in order to cover a wider range of experimental possibilities, only available in the latest version of the FDBM,^25^ motivates our decision to use the FDBM@PPM implementation.

Regarding the relaxation of the CO molecule tilting, the approach from the original PPM is maintained. For each tip position, the CO molecule is relaxed via a torsional spring leading to a contribution to the total energy given by
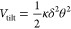
4where θ is the usual polar angle of spherical coordinates, δ = 302 pm the lever arm (distance from the outmost Cu atom of the tip to the O atom of the CO probe), and κ a torsional spring constant that can be tuned to achieve a better match with the experimental images. Notice that, instead of performing a direct minimization of the CO tilting taking into account all possible orientation changes of its charge density, a more efficient approximation is performed, which is valid for small angles. The total energy is minimized for CO displacements in the sphere determined by the oxygen probe pivot with respect to the initial position of the apex, while considering the potential energy surface determined for the rigid case. The tip is moved toward the sample in steps of length Δ*z* = 0.1 Å. In each of these steps, the tip is relaxed until the total interaction force is less than 10^–6^ eV/Å. The **F**_*z*_ curves are calculated by a numerical differentiation on the *z* position of the tip. The frequency shift is numerically calculated for the different amplitudes following ref (40) and using values for the resonance frequency *f*_0_ and the cantilever spring constant suitable for a qPlus sensor.^34^

### DFT Calculations

The simulations for both the electronic charge density and electrostatic potential of each structure and the charge density of the CO molecule acting as the tip are based on DFT following the implementation provided in the Vienna Ab initio Simulation Package (VASP) code.^41,42^ An energy cutoff for the plane–wave basis set of 400 eV was used in combination with pseudopotentials constructed after the PAW method.^43,44^ The Perdew–Burke–Ernzerhof functional^45^ was chosen to reproduce the electronic exchange and correlation, supplemented by the Grimme D3 semiempirical correction^38^ to account for the dispersion interactions.

Both the electronic charge density and electrostatic potential of each structure and the charge density of the CO molecule acting as the tip were calculated using VASP. We rewrite VASP outputs into xsf formats with the xsfConvert modular code in order to use them in the PPM suite of codes.

## Results and Discussion

### Set of Structures

In order to obtain a sufficiently large set of molecular structures, we have carried out a massive, systematic download of the atomic coordinates of “3D conformers” available on the PubChem website,^46,47^ identifying each of these structures by the CID number associated with it on this website. Using this label, we can simplify the search of molecules, especially when two structures have the same atoms. QUAM-AFM provides a Python dictionary in which it identifies the chemical formula with the CID number of each compound.

We have filtered the molecular structures on the basis of several criteria that make them of special interest for AFM research. First, we have restricted it basically to organic molecules, discarding all other compounds that may not have purely molecular forms, like organic salts or inorganic compounds. Therefore, we have selected only the molecules containing the four basic elements of organic chemistry (carbon, hydrogen, nitrogen, and oxygen) plus some other less common elements which are still frequent on organic compounds like sulfur, phosphorus, and the halogen atoms (fluorine, chlorine, bromine, and iodine). Then, we have imposed two restrictions on the size of the molecules. On the one hand, we have discarded very small molecules, namely, those containing less than eight atoms. These molecules (i.e., water, carbon monoxide and dioxide, ammonia, methane) can be observed on AFM experiments, but they are extremely mobile and display a huge variety of adsorption configurations due to their small sizes. Therefore, they are not good candidates to be identified solely by means of AFM. In addition, we have discarded very large molecules, having a structure that does not fit into a square-based cell with a side length of 24 Å. We have imposed this restriction for two reasons. Larger unit cells will dramatically increase the computational cost, and we want to use the same unit cell for all the molecules in order to avoid either the repetition of the calculation for the CO tip in each different unit cell or a cumbersome process of padding to adapt the tip calculation for a small unit cell to larger cells. The largest molecule in QUAM-AFM has a total of 85 atoms.

Although these restrictions may seem to be strong, our criteria leads to a large, representative set of molecules that includes aliphatic, cyclic, and aromatic compounds. In particular, we can find a large number of hydrocarbons (e.g., alkanes, alkenes, alkynes) together with all the traditional organic families (e.g., alcohols, thiols, ethers, aldehydes and ketones, carboxylic acids, amines, amides, imines, esters, nitriles, nitro and azo compounds, halocarbons, and acyl halides.). Besides being especially appropriate for AFM characterization, this set is particularly relevant for on-surface chemistry,^12^ a powerful alternative to traditional synthesis methods based on solution chemistry that constitutes a very active research field.

The use of deep learning techniques for AFM image-based molecular identification has to face two main challenges that are intrinsic to the technique: how to achieve chemical identification at the single atom level,^32^ and how to deal with markedly nonplanar structures.^31^ For this reason, we have restricted our selection for the database to quasi-planar molecules, that is, molecules which display only height variations up to 1.83 Å along the *z*-axis. This distance is larger than 1.5 Å—the descriptor used in ref (31) as the height range where structural information can be retrieved from 3D structures with a collection of AFM images taken at different heights—in order to include aliphatic chains with *sp*^3^ carbon atoms (methyl groups). In spite of the restrictions, we are still left with a huge data set of more than 685,000 molecules, significantly larger than those used in previous deep learning works in the field^26−29,31−33,48^ and, more importantly, that spans relevant structural and compositional moieties in organic chemistry and, particularly, in the field of on-surface synthesis.

AFM experiments are conducted on adsorbed molecules, whose structures and electronic properties may be affected by the interaction with the substrate. The molecular geometries that we use for the calculation of the theoretical AFM images are those provided by the PubChem repository that correspond to the gas phase structure of the molecules. These structures have been obtained following a sophisticated procedure based on first-principles calculations to find out the most stable configuration.^46,47^ Given the quasi-planar nature of the molecules included in QUAM-AFM, we could expect no major changes in the adsorption configuration with respect to their gas phase geometry. However, some effects like steric hyndrance, that contribute to the 3D structure in the gas phase, are most likely overcome by the interaction with the surface, even in the low reactive substrates mostly used for AFM experiments. Moreover, AFM images are very sensitive to height differences. The observed features are basically a convolution between the atomic height distribution of the molecule and the charge density and electrostatic potential at the plane where the probe is sensing.^3,4,14,16^

The study of the possible adsorption configurations of all the molecules included in our data, even considering only one substrate, is a formidable task that would require the use of massive computational resources and an incredibly long time. This fact precludes the building of the image collection directly from the optimized structures for molecules adsorbed on real surfaces. In principle, this appears to be a limitation. However, the use of a particular substrate may lead the deep learning models to specialize too much and lose the ability to generalize and identify the molecule adsorbed on a different substrate. On the other hand, the range of AFM operational parameters used to simulate the images generated for each of the molecules (see below) may introduce enough variability during the training to allow the model to identify the molecule, despite the differences introduced by the substrate.

### Operational Parameters for AFM Simulations and Effects in the Image Contrast

Although experimental AFM images reflect the inner structure of the molecules, there are a number of different operational settings that lead to variations in the contrast observed on the resulting images. In the case of constant-height AFM images, the most relevant settings are the tip–sample distance, the cantilever oscillation amplitude, and the CO tilting stiffness. While the first two can be freely chosen in order to enhance different features of the image, the last one gives rise to differences in the attachment of the CO molecule to the metal tip that are routinely observed and has been characterized in the experiments^34,49,50^

**Figure 3:**
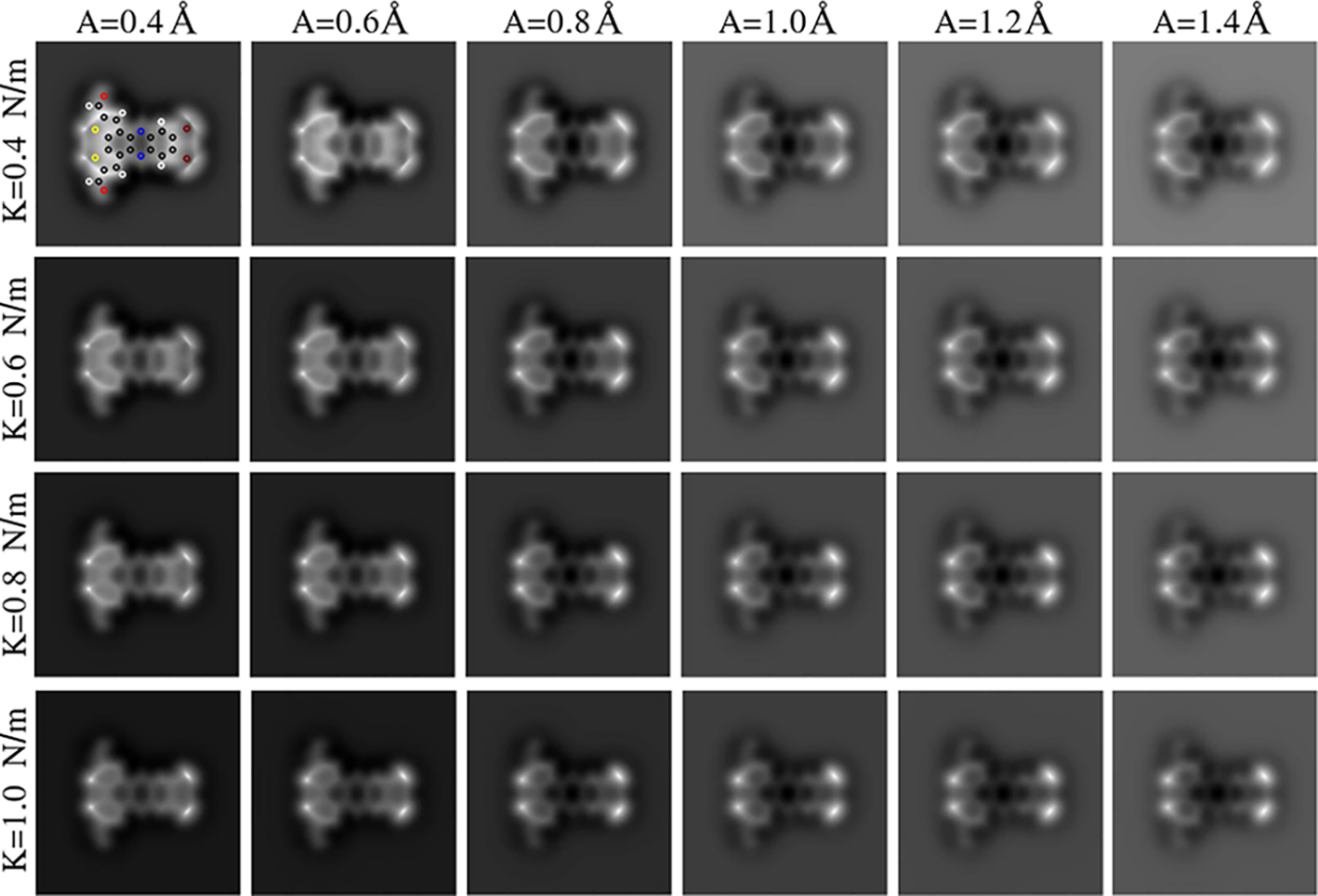
AFM frequency shift images for the 16,17-dibromo-5,8-dithia-13,20-diaza-pentacyclo[10.8.0.02,6.07,11.014,19]icosa-1(20),2(6),3,7(11),9,12,14,16,18-nonaene-4,9-dicarbaldehyde molecule (C_18_H_6_Br_2_N_2_O_2_S_2_, 134954053 CID) at a 3.2 Å tip–sample distance and for the different combinations of cantilever oscillation amplitude (A) and tilting elastic constant (κ) selected to generate QUAM-AFM. The positions of C (gray), Br (maroon), O (red), S (yellow) N (blue), and H (white) atoms are depicted in the first panel.

In order to incorporate these variations in QUAM-AFM, we have considered four values of the elastic constant describing the tilting of the CO tip (0.40, 0.60, 0.80, and 1.00 N/m), six different oscillation amplitudes of the cantilever (0.40, 0.60, 0.80, 1.00, 1.20, 1.40 Å), and 10 tip–sample distances (2.80, 2.90, 3.00, 3.10, 3.20, 3.30, 3.40, 3.50, 3.60, 3.70 Å) measured from the nearest position of the tip and the arithmetic mean of the *z*-coordinate of the atoms in the sample. We have simulated images for each molecule using the 240 parameter combinations. As we have included over 685,000 molecules in the database, our image collection contains a total of 165 million gray-scale images with a resolution of 256 × 256 pixels, which implies a computational cost of approximately 2.5 million hours. In Figures 3 and4, we provide some examples to illustrate how these operation parameters modify the AFM contrast.

**Figure 4:**
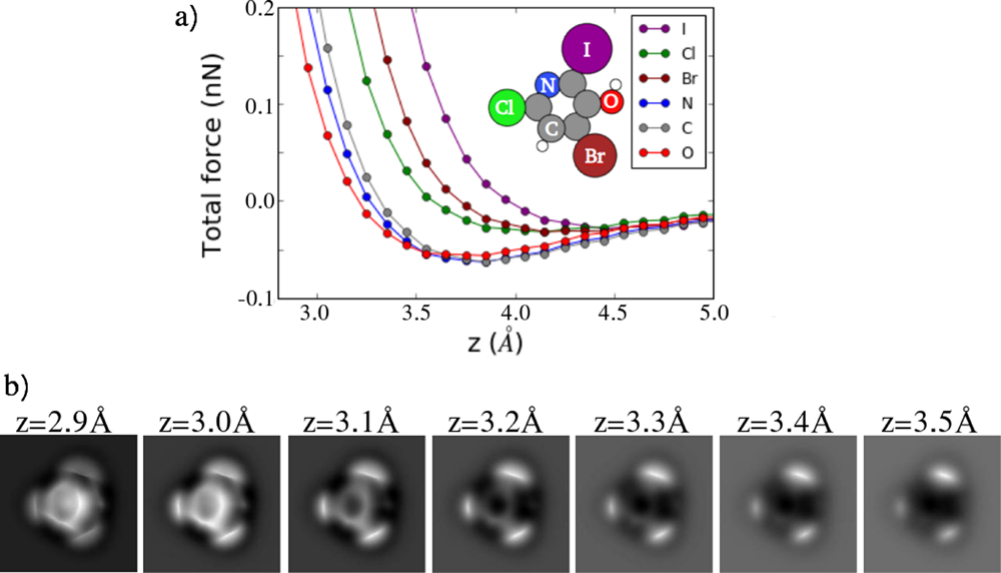
(a) Static DFT calculations of the CO molecule total force for a 4-bromo-6-chloro-2-iodopyridin-3-ol molecule (C_5_H_2_BrClINO, 53485459 CID) at Cl (green), C (gray), Br (maroon), O (red), I (purple), and N (blue) sites as a function of tip–sample distance. The inset shows the molecule structure. (b) Simulated AFM images of the structure shown in (a) at seven different heights, where the oscillation amplitude of the cantilever is 1.0 Å and the tilting elastic constant of the CO tip is 0.4 N/m.

As we have pointed out, the oscillation amplitude of the cantilever is one of the factors that can slightly modify the image appearance, as shown in Figure 3. Another factor with a remarkable influence on the images arises from the tip functionalization. Although a CO-terminated tip is routinely used in experiments worldwide, the particular adsorption configuration of the CO molecule on the apex could differ between experimental sessions and produce some differences on the final images.^51^ In order to reflect these possible variations, we have calculated the collection for several CO torsion stiffness, as can be seen in Figure 3. Notice that, as expected, softer apexes increase the lateral shifts created by the potential energy surface (PES) of the sample.^15^ Thus, for this case, their images show an apparent larger molecule.

However, the most important operation setting is the tip–sample distance. The intramolecular image contrast appears and rapidly evolves along a range of a few tens of picometers as we illustrate with the following example. Figure 4(b) shows the simulated high-resolution atomic force microscopy (HR-AFM) images of a 4-bromo-6-chloro-2-iodopyridin-3-ol molecule at seven different tip–sample distances. At larger tip–sample separations, only the halogen atoms can be seen, while upon tip approach the inner ring is unveiled. Notice that the halogen atoms appear in the images as bright ovals, which are brighter and larger the heavier they are, as has been experimentally observed.^24^ This is due to the so-called σ-hole,^24^ an effective positive charge created on halogens covalently bonded to other atoms.^53,54^ Notice that the inner ring is not imaged as a near regular hexagon. This is a consequence of the charge distribution of the molecule that creates a particular ES potential that distorts the interaction felt by the CO molecule.^15,16^ This distortion is enhanced by the CO tilting.^15^ Changes on the brightness contrast are also observed. The behavior of this image set can be explained by the different CO–sample interactions when the probe is placed on different atoms. Figure 4(a) shows the evolution of the static tip–sample forces with respect to the distances at different sites. The bigger atoms (in this case I, Br, and Cl) show larger Pauli repulsion with CO. Regarding oxygen and nitrogen atoms, although they accumulate a larger amount of charge compared to the carbon atoms, the charge density is more localized and confined, leading to a smaller charge density at some of the larger heights and to smoother variations due to the smaller repulsion.^16^ These effects are responsible for the fact that the oxygen atom is barely visible on the images. This example shows the relevance of providing a complete 3D AFM map (sets of images performed at multiple tip–sample distances) to fully describe the AFM contrast of a molecule.

**Figure 5:**
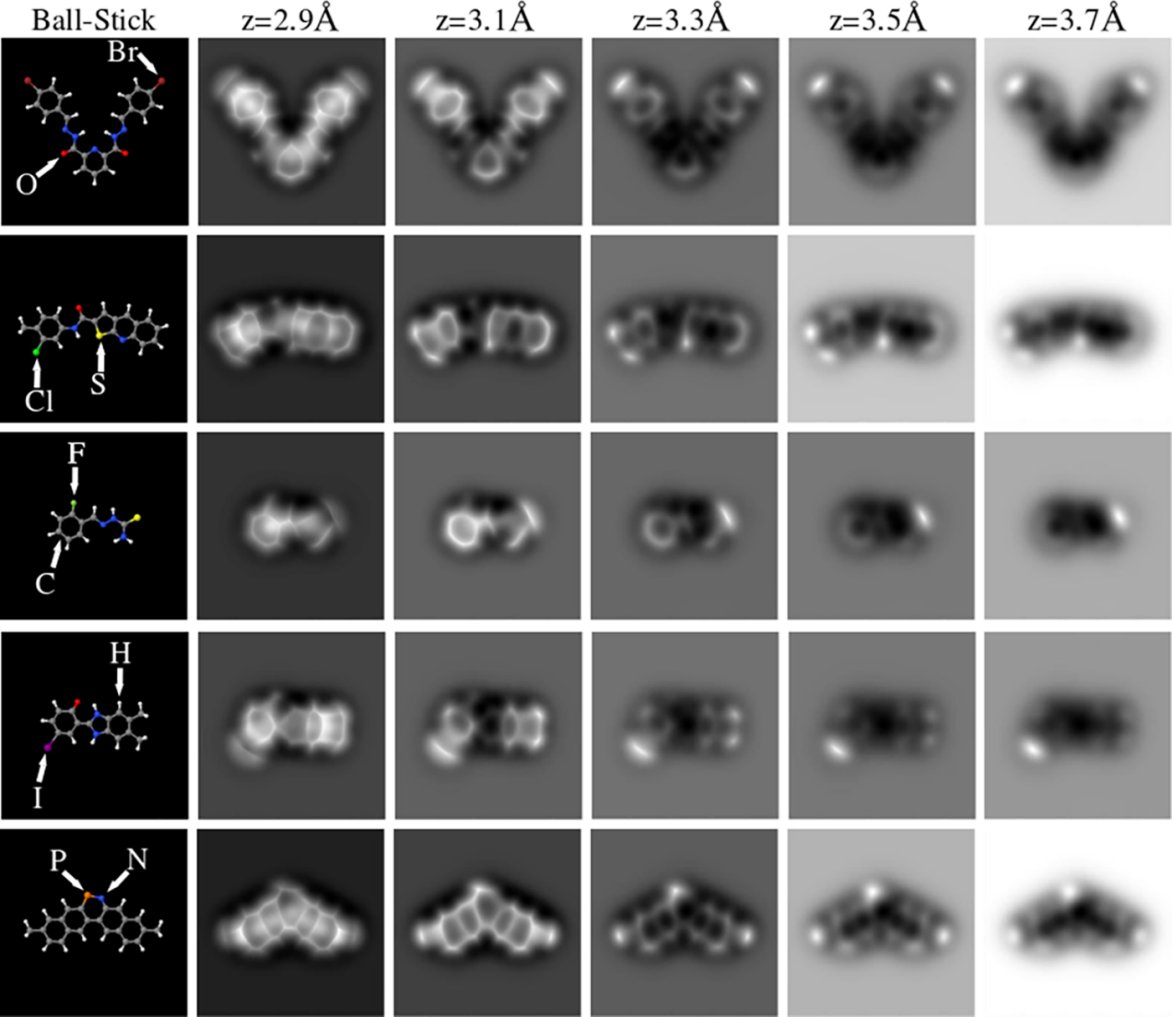
Ball-and-stick depictions performed with Jmol^52^ and their respective AFM simulated images at five different tip heights for 2-N,6-N-bis[(4-bromophenyl)methylideneamino]pyridine-2,6-dicarboxamide (2288433 CID), N-(3-chloro-4-methylphenyl)thieno[2,3-*b*]quinoline-2-carboxamide (100197613 CID), [(2-fluorophenyl)methylideneamino]thiourea (1655843 CID), 6-(5,6-dimethyl-1,3-dihydrobenzimidazol-2-ylidene)-4-iodocyclohexa-2,4-dien-1-one (107731929 CID), and 7,18-dimethyl-12-aza-13-phosphapentacyclo[12.8.0.02,11.04,9.016,21]docosa-1,3,5,7,9,11,13,15,17,19,21-undecaene (58827689 CID) molecules.

The molecule shown in Figure 4 has been simulated on a completely flat configuration where all atoms are at the same height. Therefore, it constitutes a good example since all the contrast changes are associated exclusively to the chemical properties of the molecule, determined by its composition and structure. However, experimental images involve the presence of a substrate that interacts with the target molecule and induces some internal deformations and/or a tilting of the whole molecule. As a result, in the final adsorption configuration, the atoms are at different heights. As the tip–sample interaction is so sensitive to the distance and to the molecular site that the tip is approaching (Figure 4(a)), this nonuniform height distribution could lead to AFM images with contrast changes, hampering the identification. However, 3D force maps, as those described here, provide enough information to overcome this issue. Thus, it is possible to unveil a larger number of structures in spite of the slight deformations usually found in experiments depending on the substrate. Our selection of quasi-planar molecules also contributes to minimize the substrate effect since their final adsorption configurations are rather similar and retain the main AFM features associated with their chemical groups regardless of the substrate. These features have a very characteristic distance dependence that is well represented in our data set by the 10 different constant-height images stored for each molecule and for all the combinations of operational parameters. With this information, small structural distortions induced by the substrate do not hamper the possible identification of the structure and composition of the molecule. This point is further illustrated by the examples shown in Figure 5. Lastly, it is worth mentioning that experimental images may display some distinctive features depending on the tip termination or experimental conditions during measurements. This point should not be a severe drawback to employ machine learning methodologies as we have already shown in our previous work, where we achieved very accurate automatic classifications of 60 different molecules from their theoretical and experimental AFM images.^32^

### Molecular Descriptors

**Figure 6:**
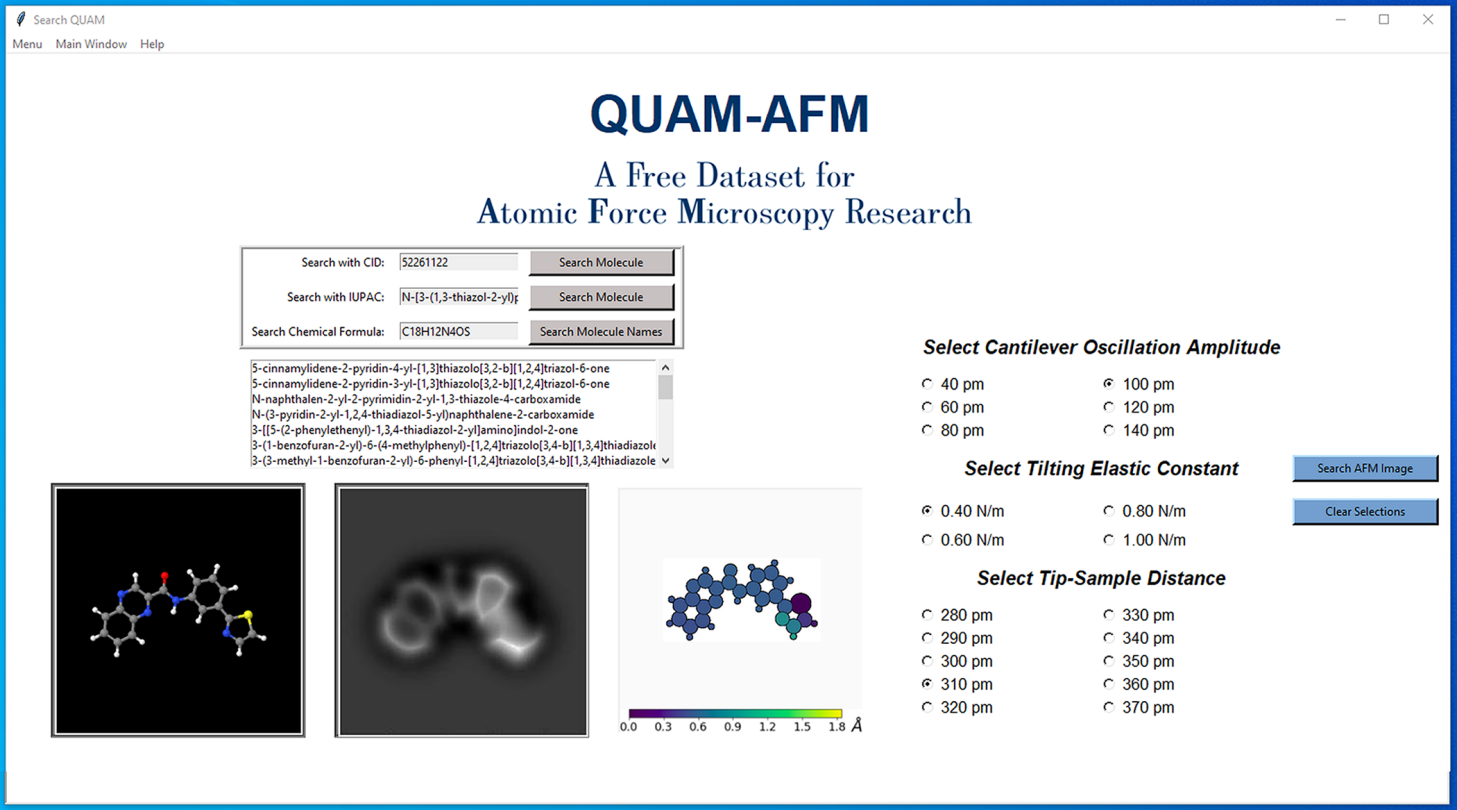
QUAM-AFM GUI developed to assist in the search of AFM images. The GUI offers different search options: CID number, IUPAC name, or chemical formula. In this last option, the GUI displays the list of IUPAC names in the database consistent with the given chemical formula to facilitate the comparison between similar molecules. Below, the ball-and-stick model and height map of the molecule are shown, together with one of the associated AFM images, corresponding to the combinations of operational parameters selected using the buttons on the right side of the screen.

One of the main problems to be tackled in deep learning is the definition of the descriptor to be used as model output. That is, in addition to a set of images to feed the model during training, it is necessary to coherently define the output and the way in which the model will provide it. As mentioned, QUAM-AFM is not only a source of information for the AFM but is designed to be used to train deep learning models and facilitate research in this area. To this end, QUAM-AFM contains data that enable multiple training possibilities. Some of the possible molecular descriptors provided by QUAM-AFM include the IUPAC names, ball-and-stick depictions, chemical formulas (that provide information on chemical species but not on the structures), and height maps (Figure 6), in line with the descriptor defined in ref (31).

It is worth noting that although we focus on explaining the molecular identification through AFM imaging the reverse problem remains open. That is, AFM images can be used as a descriptors of models to develop new AFM simulation methods.

Finally, the identification provided by the CID number of each molecule allows the set of descriptors to be augmented with information such as SMILES chains or even molecular weight by searching the PubChem website or using the open contribution Python package PubChemPy.

### Data Organization and Indexing

Managing QUAM-AFM is a key issue. As we have used the PubChem repository to extract the molecular structures, we use the CID number provided in PubChem to identify and label each molecule included in our database. This identification allows a quick search of either the corresponding AFM images, compositions, IUPAC names, or ball-and-stick depictions.

QUAM-AFM is distributed in 24 subsets, each identified by a combination of parameters; i.e., all simulations with the same cantilever oscillation amplitude and tilting of the CO tip (Figure 3) are in the same directory. Furthermore, each folder contains subfolders that identify each compound by the CID number. Therefore, each one of these subfolders contains 10 AFM images corresponding with the 10 tip–sample distances (Figure 4). This structure allows one to define a path to read each image and modify it based on a couple of conditions, which is an unbeatable layout for fast computational reading.

The identification by CID number together with the distribution of the data in neat folders make QUAM-AFM very accessible for artificial intelligence (AI) problems. That is, instead of generating three subfolders for the training, validation, and test subsets,^37^ it is enough to create three lists with the CIDs belonging to each subset.

### GUI to Easily Scan the QUAM-AFM Data Set

QUAM-AFM is ideally suited to train deep neural networks. Nonetheless, its applicability goes beyond predictive models. An AFM image repository of this magnitude can be used as an immediate source of information for those researchers that use AFM techniques, removing the need for extra simulations which are computationally expensive. Unfortunately, due to the large amount of data contained in this database, it is not feasible to apply the usual shell commands to search for images, let alone browsing by opening directories. For this reason, we recommend a different method to access the images in accordance with the task at hand.

We have developed a fast user-friendly GUI (Figure 6) to search for AFM images of a specific molecule with particular parameter combinations. This interface has been developed in Python 3.7 with dependency on the tkinter, pickle, shutil, and pillow packages^55^ and successfully tested on Linux (Ubuntu 18.04, Kubuntu 18.10, and Centos 7), Windows 10, and macOS (for High Sierra and later versions). The Python code, *QUAM.py*, is included in the folder named GUI in the data set distribution (see the Data and Software Availability statement) and can be launched simply by the command “*python QUAM.py*”. As shown in Figure 6, it is possible to search for molecules interactively in three different ways: (i) The first search method is the PubChem CID number because QUAM-AFM associates each structure with this identifier. (ii) Searching by IUPAC name is also included. Complementary to the CID number, each set of AFM images of a molecule on the collection is indexed also with the IUPAC name, the most widely used method to name molecules. This nomenclature was developed with the aim of strictly identifying any molecule and is based on a specific language that constructs the names of each molecule from a parent chain to which prefixes and suffixes are added to identify the type of functional groups. (iii) It is also possible to search for molecules by chemical formula. This last search option displays the list of IUPAC names of the molecules in the data set that are consistent with the given chemical formula. This feature makes it possible to visualize similar structures, scrolling the list and selecting the IUPAC name with the mouse. All these search methods complement each other simultaneously so that when searching by one of the identifiers the rest are filled in automatically. Once the structure is found, the operational parameter buttons allow the user to perform a quick search for a specific AFM image.

The search engine consists of a series of Python dictionaries where the key or value is the CID number, which is used to identify each of the image folders. In this way, when searching for the IUPAC name, the search engine modifies it by the CID number, calling the image update functions with it. The search by chemical formula is carried out in a similar way, although in this case the displayed list uses the chemical formula as key and all the IUPAC names associated with it as values which, in turn, are used to look up the CID number in the corresponding dictionary. Once the target structure has been found, the search for a specific AFM image is performed with a series of buttons that allow the selection of specific simulation parameters. These parameters are used as the key of a dictionary whose value is the directory in which the image is stored. This identification renders the selection of AFM images, height maps, and ball-and-stick representation computationally efficient. Finally, the user has the option of copying the images (or entire directories) to the selected address, saving the results found.

## Conclusions

In this work, we have presented QUAM-AFM, a free scientific data set generated from a selection of more than 685,000 organic molecules that includes the most relevant compounds of organic chemistry. The data provided for each molecule consist of a broad set of AFM image simulations, ball-and-stick depictions, IUPAC names, and chemical formulas. The operational parameters selected to generate the AFM images for each molecule are six cantilever oscillation amplitudes and four values of lateral tilting stiffness. Additionally, we have performed the simulations with 10 tip sample distances for each combination of parameters. Consequently, QUAM-AFM contains 240 AFM simulations from each molecule, resulting in a total of 165 million images with a resolution of 256 × 256 pixels. In order to simplify the use of the collection, we have developed a graphical interface that allows the search for structures by CID number, IUPAC name, or chemical formula. Therefore, comparisons among similar structures or assessments of the effect of different operational parameters in the AFM images are fast and user friendly.

The results presented here can be of great interest for the AFM community both as a comprehensive reference to illustrate the AFM contrast associated with the most relevant chemical moieties in organic chemistry and as a data set suitable to support theoretical investigations in this field. In this sense, QUAM-AFM is a pioneering data set as it provides not only 2D images of a very large number of molecules but 3D image stacks for each one, spanning the distance range where tip–sample interactions vary significantly. The particular design and organization and the large amount of information stored in the data set make it especially suitable to proceed with machine learning or AI techniques applied to AFM images in order to automatically extract information about the structure and composition of molecules. Moreover, we expect QUAM-AFM to be a source for further work in different aspects of AFM characterization and a platform to generate new research lines within this technique.

## Data and Software Availability

QUAM-AFM requires 713 GB storage and has been published, with free access for downloading.^56^ For testing purposes, we have generated QUAM-AFM Lite, a reduced version of QUAM-AFM with a set of 1755 molecules (2 GB storage in total) together with the graphical user interface and all its functionalities, it is also freely accessible and downloadable.^57^

## Acronyms

DFT: density functional theory
AFM: atomic force microscopy
GUI: graphic user interface
PPM: probe particle model
FDBM: full-density-based model
AI: artificial intelligence
